# Indents

**Published:** 1912-07-15

**Authors:** 


### INDENTS.

**☞Brief Articles of Technical or Scientific Value will receive notice and comment. Contributions invited.**

*The Canadian Oral Prophylactic Association.* In some respects this is a unique organization, and it would seem worth while to call attention to its objects and to the work which it is aiming to accomplish. It was organized by several of the dentists of Ontario for the purpose of improving conditions relative to the prevention of dental diseases among the people. First, it began a study of tooth powders and pastes on the market with the idea of recommending those most suitable. In this connection we do not believe that all of the factors entering into the use of tooth powders or pastes in the different mouths have been sufficiently analyzed by the profession on the basis of laboratory and bacteriological experimentation, or the effects on the fluids and tissues of the mouth. This is something which should be scientifically made out and published, so that the profession and the manufacturers will be able to meet the situation in an intelligent and humanitarian way.

After looking into the various products on the market the Association decided to have a powder manufactured for itself from a formula which was to be published. Then a study was made of the proper form and size of tooth brush for the different surfaces of the teeth, and this resulted in several forms of brushes which more nearly fulfil the function for which they were intended than any we have seen.

The chief difficulty was to get chemists to manufacture the products up to standard grade and to keep them up. So far as the brushes were concerned no prominent firm seemed to care to undertake their manufacture. But the officers of the Association kept working away at the various problems till they finally mastered them. They insisted on chemists keeping their products up to formula specifications, and they finally got their brushes made. The demand has now become so great that the very firms which originally declined to manufacture them are clamoring for this privilege.

The Association has its products marketed to the people at a very reasonable figure and yet there is a small profit on every article that goes out, which comes to the Association. And here is the significant thing in the movement. Every dollar so earned is spent not for the profit of the members of the Association, but for educational and philanthropic purposes. Free public lectures on the importance and care of the teeth are given and the expenses paid by the Association. Pamphlets by the thousands are distributed to the public, charts and drawings are loaned to societies and to individual lecturers and a general campaign of education is carried on through various channels. Last, but not least, the Association plans to create a fund for the care of members of the profession who through old age or misfortune are incapacitated for work and are without the necessary means of support. We have never known of another organization quite like this in the dental profession and it will be interesting to study the outcome of the movement and to note how far reaching it may eventually become.—C. N. Johnson in *Review*.

*Infusion Anesthesia.* F. Rood, in the British *Medical Journal*, presents a series of twenty-one cases in which ether was administered by intravenous infusion in a 5 per cent solution in normal saline. This method was originated by Burckhardt. The mode of inducing anesthesia is as follows: An hour before the induction a hypodermic injection is given, which consists of scopolamine, 1-100 grain, morphine, 1-6 grain, and atropine, 1-100 grain. The patient is placed on the operating table and the selected arm lightly bandaged to a splint to prevent flexion of the elbow; a few drops of eucaine having been injected into the skin, the vein is exposed through an incision of a third of an inch. The cannula having been tied in, it and the wound are packed round with sterile gauze. The strictest asepsis is, of course, preserved throughout this procedure. The solution is run in rapidly during the period of induction, which usually lasts about ten minutes. It is usually possible to attain surgical anesthesia with the use of 8 ounces of the solution, while for the maintenance of the anesthesia as little as a pint an hour (1 ounce of ether) is in most cases enough. The most striking features of infusion anesthesia, the author maintains, are: (1) Extreme flexibility so that the condition of the patient can be adjusted with great nicety and can be changed in the direction of deepening or lightening of anesthesia with really remarkable rapidity. (2) The patient begins to come round very soon after the anesthetic is stopped and is able to converse rationally within a few minutes. (3) Post-anesthetic vomiting and pulmonary irritation are both extremely rare.

*A Reason for Inefficiency.* A child came to my office with his mother and made an appointment for dental services. He arrived twenty minutes late for his appointment and when he was taken to task about it, said, "One of the boys said I made a face at him and my teacher kept me in for it, that is why I am late." I told him that he should not make faces and he said, "I just can't help it." I said nothing more, and upon examination of that child's teeth saw plainly why he could not refrain from making what his class-mates call faces. I found in the two first lower molars large occlusal cavities, each containing a hypertrophied pulp rising at least 1-16 of an inch above the tooth, which forced the child to always keep his jaws apart, using only his anterior teeth to nibble with, so to speak. At times when bringing the jaws into occlusion the pain from these pulps would cause him to jump and squirm, which the other children considered "making a face." I asked the boy how he managed to eat. His reply was that he only ate soft things that he could bite on his front teeth. I next asked him how he was getting along at school and was told that he was always kept in and nearly always last in his classes. This condition had existed for months. In a short time I had relieved the conditions and attended to the teeth properly. He returned to me in six months for other services and in that time he had developed into a strong vigorous boy. He stated that he was never kept in and had made such progress in his studies that he had jumped two grades, which was considered good work. This case in itself shows the importance of dental hygiene in schools. Had there been a requirement for the examination of children's teeth this ward of the city could have completed his education much sooner.—Dr. W. M. Bartlett in *Review*.

*Just a Hint on Inlay Work.* After the successful treatment of a dead tooth and its test for a reasonable time—I substitute an inlay for the plastic filling and take the precaution to melt a hole through the center of the wax impression to the floor of the cavity. I then cover the upper end of the opening with a thin layer of wax. When the inlay is completed, the orifice on the under surface of the inlay may be enlarged.

This serves as an extra anchorage, when filled with cement.

This procedure in case of a recurrence of pain, enables the dentist to quickly run a bur through the thin layer of gold above—thereby considerably lessening pain and additional discomfort to the patient—which otherwise would follow, if the solid gold had to be perforated. Furthermore, the danger of displacing the inlay is lessened.—W. Lowenthal in *Digest*.

*Polishing Plates.* Add a little glycerine to the pumice instead of water, and it will adhere to the cone and plate better and give quicker results.—I. L. Porter in *Digest*.

*Nerves in Dentine.* On Thursday, February 1, Mr. Howard Mummery read before the Royal Society his long-expected pronouncement upon the nervous distribution in dentine. After years of patient research, he has finely and conclusively demonstrated the existence of nerve fibrils in the tissue. It is proved by hundreds of sections and dozens of different staining methods and must now pass into current teaching. The effect of the discovery clears up many incongruities and once more brings our anatomy into line with common sense and the natural order of histology throughout the body. Mr. Mummmery has added to our debt of gratitude towards him, a debt already large.—*Dominion Journal.*

*To Solder a Richmond Crown.* To flow Richmond Crown Solder—with a pointed fissure bur put hole through investment at labio-gingival of crown to be soldered and it will flow up the edge of joint perfectly. —I. L. Porter in *Digest.*

*Somnoform.* To The Editor: Please inform me about Somnofoım and its effects when used as an anesthetic in extracting teeth.

Last November, when my wife had some teeth extracted our dentist spoke very highly of this preparation as without bad effects. It left her, however, with a very irritable asthmatic respiratory tract and the circulatory system has been upset, causing a congestion in all the organs, with the symptoms which accompany such conditions. It seems as if the pressure brought to bear on the heart during her severe attacks of coughing and difficult breathing caused the heart to dilate, which made the tricuspid insufficient.

She has been in bed the greater part of the time since December 18, when her heart seemed to give way under the strain. She is able to be up and around now, but that is all; on slight exertion she loses her breath. She was a strong, healthy woman of a nervous temperament, somewhat stout but solid. Sugar was found in the urine, which quickly cleared up on diabetic diet and has not returned though carbohydrates are allowed in moderation. The glycosuria was supposed to be due to the shock. I would like to know if any similar experiences have been reported.

B. R.

Answer.—Somnoform is said to be a mixture of ethyl chlorid 60 parts, methyl chlorid 35 parts and ethyl bromid 5 parts. The relative value of ethyl chlorid and methyl chlorid as compared with ether and chloroform is still an open question, as also is the relative safety of administration. In a general way they have been found to stand, both in effect and safety, between nitrous oxid and ether.

W. J. McCardie (*Brit. Med. Jour.*, March 17, 1906, p. 616) cites a number of fatalities from the use of ethyl chlorid and also states that several deaths have occurred from Somnoform. In a former note in this department (*The Journal*, Nov. 21, 1908, p. 1788) it is stated that eighteen deaths had been reported from ethyl chlorid; but the number of administrations is not accurately known, so that the mortality rate cannot be stated positively. So far as we know, symptoms similar to those given by our correspondent have not previously been noted as resulting from the use of ethyl chlorid, or from the other alleged ingredients of Somnoform.—*Journal A. M. A.*, March 16, 1912.

*Protecting Gum Margins.* To protect the gum margins around a root prepared for a cast Davis crown between sittings, I use a small piece of gutta-percha sufficiently warmed to make plastic. Thoroughly dry the root end and apply the gutta-percha partially, moulding it to place. To insure the permanency of this covering until the next sitting I use a very small sized tack. Warm it in the flame and imbed it through the gutta-percha into the root canal. Your temporary protection will stay until you remove it yourself.—A. E. DeRiemer in *Review*.

*Cast Metal Appliances.* The author offers the results of extensive experiments with the casting method of prosthetic appliances, and briefly states his conclusions as follows:

Gold plates cast under pressure are superior to swaged ones in regard to faithful reproduction of the model, adaptation in the mouth, and resistance to the stress of mastication. Prolonged observation of such plates being worn in the mouth has fully demonstrated the advantages of the casting method over any other. Cast aluminum plates may be advantageously employed owing to their great lightness, their favorable behavior toward the oral mucosa, which is not irritated by this metal, and by the ease with which they can be cleaned by boiling; finally, by the possibility of combining an aluminum base-plate with vulcanite without the risk of the metal being acted upon by the hydrogen sulfid from the rubber. Commercial aluminum may be employed if it is correctly manipulated, though 99.8 per cent. pure aluminum specially prepared for our purpose is far preferable. Pure aluminum plates keep very well in many mouths, and can be successfully combined with iridio-platinum clasps. Contrary to general opinion, platinum-pin teeth can be employed without the platinum undergoing so much alteration as to impair the solidity of the appliance.

The general adoption of cast metal appliances in the future seems assured, and their superiority over those made by the old methods is indisputable. Yet while seemingly easy, the casting method requires special training and great care to yield perfect results.—Paul Martinier in *Cosmos.*

*Symptoms of Nasal Obstruction.* Briefly enumerated the signs and symptoms to be looked for are as follows: Most frequent and most characteristic is mouth breathing during all or part of the time, with noisy respiration or actual snoring at night. Restlessness during sleep with a tendency to awaken and call for water to relieve the extreme dryness of the mouth. Advanced cases will show a characteristic face, easier to recognize than to describe, with blunted mental activities, backwardness at school, from no apparent cause, and more rarely a history of nocturnal enuresis, or night-terrors, may be developed. Chronic cough, which is slightly productive and which becomes worse in winter and during periods of damp weather is present as a rule.—Dr. Faught in *Items*.

*Controlling Alveolar Hemorrhage.* Soften gutta percha and press it into the alveolar socket with a considerable excess. Have the patient bite into it to occlusion, and force the excess against the buccal and lingual surfaces. Take out and cool and trim away such portions as are not needed and make smooth. Replace and let the patient hold in place by closing the teeth. It makes an effective mechanical plug, which may be of use in retaining medicinal agents.—Dr. E. A. Bogue, *Journal.*

*Protecting Metal Plates During Vulcanizing.* When a base plate of aluminum or Watts metal, cast or swaged, is used in plate work, it may become tarnished by the sulphur escaping in vulcanizing, making it difficult to regain the lustre. This may be avoided by painting the exposed metal surfaces with shellac or sandarac, which will protect the metal from the sulphur and greatly facilitate the polishing of the metal part.—M. L. Schmitz in *Review*.

*Use of Formaldehyde in Dentifrices.* Formaldehyde is recommended for use in dentifrices where positive antiseptic effect is to be produced, but its irritating effects render a satisfactory preparation difficult to manufacture, says the American Druggist. A French pharmacist reports that the use of menthol in the dentifrice not only masks the odor of the formaldehyde, but also neutralizes its irritating effects. A saponaceous basis with about 20 per cent. of alcohol is recommended as giving most satisfactory results, saccharin or glycerin being used for a sweetening agent. As a flavoring agent a combination of menthol, oil of wintergreen and vanillin may be used. In making liquid dentifrices containing formaldehyde care must be observed not to use an excess of soap, as in course of time the formaldehyde will cause a precipitation, or, in extreme cases, even cause a coagulation with certain soaps. Pure castile soap is the best soap to use in all dentifrices, liquids, paste or powder.—*American Perfumer.*

*A Perfectly Fitting Porcelain Inlay.* A porcelain inlay which fits the cavity exactly and leaves a minimized cement line may be constructed in the following manner:

An impression of the cavity is taken with dental lac, which is then embedded in moldine in the cup of a swaging outfit, and the gold or platinum matrix is swaged by the lac model. Oiling the lac model makes the removal of the swaged matrix easier.

When the foil is perfectly adapted to the lac model it is covered with a layer of investment compound, which is allowed to harden. When the investment compound has thoroughly hardened, the matrix, and its backing of investment compound, is removed from the cup and invested in the regular way, in one of the nickel trays used in porcelain work.

The advantages of this method are: (1) A model is unnecessary, and time is saved; (2) Thick foil may be used, lessening the chances of distortion in baking; (3) The matrix cannot be distorted in removal from the model or when invested in the tray; (4) The inlay fits the cavity perfectly. —C. M. Torrance, D.D.S., Frankfort-on-Main, Germany, *The Dental Cosmos.*

*The House-Fly and the Bed Bug.* One of the characteristics of the present-day campaign for the prevention of disease is the homely, practical way in which facts are being placed before the public. Many of our state boards—through bulletins—are doing excellent work in this direction. As a result, some popular ideas are being sadly shaken. The little house-fly for instance, has been for years the subject of household poetry, and has been referred to as the harmless and innocent companion of man. The bedbug, on the contrary, has been looked on with speechless aversion. He has no social standing. Even the mention of his name has not been considered good form in our best circles, while the least suspicion of a speaking acquaintance with him has been regarded with horror. In the May number of the *Bulletin of the North Carolina State Board of Health*, Dr. Cyrus Thompson, in an article on "Flies and Filth," says: "Now as a matter of unprejudiced fact, barring the sting of the bite and the odor of the encounter, the bedbug is a much more eligible companion than the house-fly, whether of bed or of board. But if bedbugs, comparatively cleanly of habit, crawled all over our plates, table and food just as the house-flies crawl, fresh from the foulest filth of every pestilential kind, who would eat or even sit at the table for a moment? I am not making a plea for the elevation of the social status of my nocturnal friend, who loves darkness rather than light but I am declaring that his deeds are not nearly so evil and destructive as those of the house fly." Put this statement before every American housekeeper, and the doom of the typhoid fly is sealed. The bedbug has been, for generations the abomination of the housewife, and the object of her unrelenting warfare. Once convince American women that the fly is more lothesome and dangerous than the bedbug, and the ravages of this typhoid-breeder and filth-distributor will be over.—*Journal A. M. A.*

*Cold Storage and Health.* In its relation to the health of the people and less directly in its effects on the cost of living, the cold storage problem is of immediate interest to the medical man. At the outset it must be admitted that no serious complaints can be brought against the cold-storage warehousemen in general on the ground of unsanitary or unscientific methods of conducting their establishments. Any abuses in this respect are exceptional; and the satisfactory condition of the plants is attested by the results of government inspection. The other questions raised have very recently been made the subject of an inquiry by a commission appointed for this purpose by the Governor of Massachusetts. The report of its five members commands notice as the latest pronouncement on this debated topic. The commission recognizes that cold storage has become a fundamental necessity in the distribution of the food-supply of the nation, and sees its principal economic function in the fact that it enables the surplus of certain products in the season of natural plenty to be carried over to meet the demand in the season of natural scarcity. The charge that cold storage in general is detrimental to public health is refuted by an impartial examination of this subject in its hygienic aspects.

In the words of the report: "While abuses have arisen, through the holding of food-products in cold storage for unduly long periods and through the handling of foods by improper methods before and after as well as during refrigeration, the benefits that have come from the salvage of food through cold storage far outweigh any evils that have developed in this field. Cold storage has brought about an expansion and diversification of the food-supply of the population, making certain kinds of food more abundant and more accessible. It thus makes for the conservation of the vital resources of the people. The gain from this source is universal and permanent; the injuries are occasional and temporary, and can be eliminated by proper regulation."

There are numerous data now available regarding the changes which certain food-products, especially meats, poultry and eggs, undergo during cold storage. These have in the main been gathered by the United States Department of Agriculture and there has been a notable improvement in the commercial handling of poultry during recent years as the result of Dr. Pennington's investigations. Cases of ptomain poisoning alleged to have resulted from the consumption of cold-storage foods are often mentioned in newspapers; but manifestly such testimony is of dubious value. It is evident that the condition of goods when they come out of storage depends very largely on their condition when they go in. The greatest care is needed in the selection and preparation of food-products for preservation; and failures in this direction should not be charged to refrigeration.

The progressive changes taking place in perishable food-products are not absolutely suspended by refrigeration. Therefore a food-product that has been held in cold storage is never just as good as the perfectly fresh article, other conditions being equal. The deteriorations first appears in a change in flavor, which may affect the palatability of the food, but does not necessarily impair its wholesomeness or nutritive value. Scientific investigation in this field has not yet furnished sufficient evidence to enable one to fix the time limits of cold storage for different commodities; for the proper period depends largely on the methods of previous handling and preparation.

The Massachusetts commission reports that "on the whole, prolongation of cold storage beyond one year, even under correct conditions, appears to be undesirable and prejudicial to the public health." It believes that there is need of legislation for the regulation of the business and proposes that extensive powers be delegated to the State Board of Health for this purpose. The main objects are to prevent improper handling of foods, undue prolongation of the storage period and deception of purchasers through the sale of cold-storage articles as fresh goods. Finally the commission believes that the influence of cold storage is to make prices lower and steadier, thus disposing of the bugaboo of speculative manipulation.—*Journal A. M. A.*

*Ward's Inlay Investment Material.*

| | | |
|---|---|---|
| ℞ | XXX Silica . . . | 16 parts |
| | No. 200 Flint . . . . . | 6 " |
| | Model Plaster . . . . | 5 " |
| | Impression Plaster . . . . | 5 " |

Thorougly mix.

*Instruments for Inserting Silicate Cement Fillings Without Risk of Subsequent Discoloration.* In inserting silicate cements fillings, for which such excellent qualities are claimed many operators have become discouraged by the discoloration of the filling following the use of blued steel or nickel-plated steel instruments. The instruments of agate, bone, or tortoise-shell recommended for this purpose, owing to the inherent physical properties of these materials, are not very satisfactory, while instruments of platinum or tantalum are so expensive as to deter many operators from purchasing them.

A simple and cheap method of making suitable instruments which allow of as easy manipulation as steel instruments and which will not discolor the silicate cement fillings, consists in soldering heavy gold or platinum foil with a suitable high-karat gold solder over the point and part of the shank of ordinary filling instruments of steel, with the needle-point flame of an ordinary blow-pipe or an orthodontia soldering blowpipe. If the foil is worn off by continued use, the soldering operation can be repeated.—R.H. Riethmuller, Ph.D., *The Dental Cosmos.*

*General Ether Anesthesia by Intravenous Route.* Lofberg expatiates on the advantages of administering the anesthetic by the intravenous route in operations on the face, neck, etc., although he does not quite agree with Burkhardt's opinion that this technic is the least harmful and most agreeable. The latter has used it in 250 cases without embolism or thrombosis, although he reports asphyxia from overdosage in three cases. Kummel has had no embolus in 100 cases and no thrombosis in his last fifty cases. Lofberg reviews the other literature on the subject and describes the technic with an illustration of it as applied in practice. He then reports his own experience with it. The operations in his three cases were for ruptured tubal pregnancy in a woman of 26, resection of the pylorus in a man of 47, and amputation of the femur for diabetic gangrene in a women of 66. The anesthesia was not quite perfect in the first two cases but the patient made a smooth recovery. The third patient went under in five minutes and the anesthesia was complete; 40 gm. ether were used, and she recovered from the operation but died nine days later from embolism. An embolus plugged the pulmonary artery and a thrombus was found in the basilical vein 4 cm. above the point of infusion and others farther up in the cephalic and subclavian veins, none anywhere else. A total of 498 cases of intravenous ether anesthesia are now on record, with two deaths, one of unknown cause and the other the case of embolism reported above, while Kuttner has reported a case of clinically diagnosed embolus. Lofberg thinks there can be no doubt that the fatality in his case was the result of the infusion, but whether the ether was responsible for it or the salt solution vehicle is not absolutely certain. He infused 810 c. c. of physiologic salt solution, containing 5 per cent. of ether.—*Journal A. M. A.*, June 29, 1912.

*Alopecia.* The following treatment is recommended by Dyer as a suitable one to be adopted in most cases. The frequent washing of the scalp with green soap or tar, resorcin, naphthol and sulphur soap, and the application of stimulating substances. Of these he mentions chloral hydrate, tincture of jaborandi, spirits of rosemary, cantharides (in tincture and guardedly), tar oils (cade, birch pine, etc.), castor oil, croton oil (in minute quantity), alcohol and chloroform. The use of any application should be intelligent; oily substances used when the scalp and hair are dry; desiccating or alcohol preparations when the hair is oily or the scalp greasy. Combinations may be made antiseptic with resorcin (2 to 5 per cent.), salicylic acid (2 to 5 per cent.), lactic acid (2 to 5 percent.), bichlorid of mercury (1 to 1,000), or with carbolic acid (not over 2 per cent.) each of which is also somewhat stimulating to hair growth. The use of the high frequency *effluve* in some cases of long standing has proved satisfactory.—*Journal A. M. A.*, June 22, 1912.

